# Impact of Nicotine on Cognition in Patients With Schizophrenia: A Narrative Review

**DOI:** 10.7759/cureus.24306

**Published:** 2022-04-20

**Authors:** Violeta Spasova, Saqib Mehmood, Amjad Minhas, Rabia Azhar, Silpa Anand, Sondous Abdelaal, Sunder Sham, Tabssum M Chauhan, David Dragas

**Affiliations:** 1 Psychiatry and Behavioral Sciences, California Institute of Behavioral Neurosciences & Psychology, Fairfield, USA; 2 General Surgery, California Institute of Behavioral Neurosciences & Psychology, Fairfield, USA; 3 Medicine, California Institute of Behavioral Neurosciences & Psychology, Fairfield, USA; 4 Family Medicine, California Institute of Behavioral Neurosciences & Psychology, Fairfield, USA; 5 Internal Medicine, California Institute of Behavioral Neurosciences & Psychology, Fairfield, USA; 6 Plastic Surgery, California Institute of Behavioral Neurosciences & Psychology, Fairfield, USA; 7 Pathology, California Institute of Behavioral Neurosciences & Psychology, Fairfield, USA; 8 Research, California Institute of Behavioral Neurosciences & Psychology, Fairfield, USA

**Keywords:** nicotinic cholinergic receptors (nachrs), nicotine, tobacco, schizophrenia, smoking, cognition, varenicline

## Abstract

Nicotine is the psychoactive component given tobacco has several main components and acts as an agonist for nicotinic acetylcholine receptors (nAChRs) in the nervous system. Although the ligand-gated cation channels known as nAChRs are found throughout the nervous system and body, this review focuses on neuronal nAChRs. Individuals with psychiatric diseases such as schizophrenia, comorbid substance use disorders, attention-deficit hyperactivity disorder, major depression, and bipolar disorder have increased rates of smoking. These psychiatric disorders are associated with various cognitive deficits, including working memory, deficits in attention, and response inhibition functions. The cognitive-enhancing effects of nicotine may be particularly relevant predictors of smoking initiation and continuation in this comorbid population. Individuals with schizophrenia make up a significant proportion of smokers. Literature suggests that patients smoke to alleviate cognitive deficiencies due to the stimulating effects of nicotine. This narrative review examines the role of nicotine on cognition in schizophrenia.

## Introduction and background

Schizophrenia is a chronic mental condition with a lifetime prevalence of about 1% in the general population [1]. The disorder is characterized by positive, negative, and cognitive symptoms. Schizophrenia can commonly result in social impairments [2]. Although the actual etiology of schizophrenia remains unknown, environment, genetics, and altered brain neurobiology may play a role [3]. Attention, executive functioning, learning deficits, linguistic knowledge, and spatial working memory are all connected to cognitive impairment in schizophrenia [4,5]. Furthermore, despite their efficacy in the treatment of positive symptoms, antipsychotics may contribute to cognitive impairment in schizophrenia [6]. Several studies have shown that schizophrenia patients have an extremely high prevalence of smoking, increased rates, and intensity of tobacco smoking of almost 90% compared to only 33% in the general population and 45-70% in patients with other psychiatric diagnoses [7,8].

The increase in nicotine receptors caused by smoking has been linked to lower levels of social withdrawal, motivational responses, blunted emotions, and improved cognitive function [9-11]. Although nicotine has been shown to improve cognitive deficits in schizophrenia, the underlying neurobiological mechanism remains poorly understood [12]. A review by Levin and Rezvani (2006) reported that nicotinic co-therapy may be a useful adjunct in the treatment of schizophrenia, potentially lowering cognitive impairment [12]. Nicotinic acetylcholine receptors (nAchRs) have emerged as a possible therapeutic target for the treatment of schizophrenia-related neurocognitive dysfunctions [8-13]. Varenicline is a partial agonist and may be a useful therapy for treating not just nicotine dependency in schizophrenia but also the cognitive deficiencies that are one of the disorder’s primary symptom clusters [14]. This review examines the effect of nicotine on cognition in schizophrenia patients.

## Review

Methodology

With the specific combination of keywords including “Nicotine/administration and dosage,” “Nicotine/adverse effects,” “Nicotine/agonists,” “Nicotine/chemical synthesis,” “Nicotine/chemistry,” “Nicotine/genetics,” “Nicotine/metabolism,” “Nicotine/physiology,” “Nicotine/therapeutic use,” “Nicotine/toxicity,” “Schizophrenia/anatomy and histology,” “Schizophrenia/chemically induced,” “Schizophrenia/chemistry,” “Schizophrenia/drug therapy,” “Schizophrenia/epidemiology,” “Schizophrenia/diagnosis,” “Neurobehavioral Manifestations/anatomy and histology,” “Neurobehavioral Manifestations/chemistry,” “Neurobehavioral Manifestations/diagnosis,” “Neurobehavioral Manifestations/etiology,” etc. an article search was conducted on Google Scholar, PubMed, MEDLINE, Scopus, and Cochrane. Search results were reviewed, and only articles describing the effect of nicotine on cognition in schizophrenia patients were selected. Articles published in the English language were included in this review. All study designs including cohort studies, case-control studies, and randomized controlled trials were analyzed in this review.

Results

A total of 1,202 articles were found in multiple databases, including Google Scholar, Cochrane, Scopus, PubMed, and MEDLINE, of which 888 were initially removed due to repetition and irrelevance. After analyzing the titles and abstracts at the first screening level, 635 articles were further removed. A total of 90 articles were included in this review.

Nicotine and Schizophrenia

**Figure 1:**
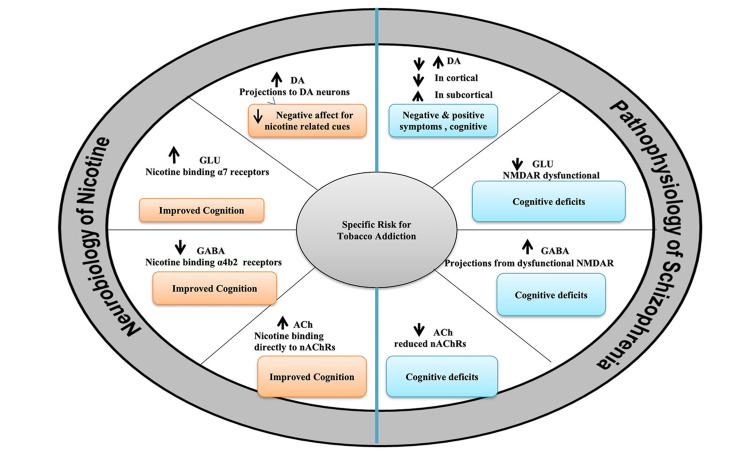
Pathophysiology of schizophrenia and neurobiology of nicotine. DA: dopamine; GLU: glutamate; Ach: acetylcholine; nAchRs: nicotinic acetylcholine receptors; GABA: gamma aminobutyric acid; NMDAR: N-methyl-D-aspartate receptors

The nicotinic system in schizophrenia attracted initial interest after reports of greater smoking rates in schizophrenia patients compared to the general population [15-17]. The pathophysiology of schizophrenia and the neurobiology of nicotine is shown in Figure 1. Several characteristics of the smoking and schizophrenia relationship indicate a potential dysfunction in the nicotinic acetylcholine receptor system, suggesting that this dysfunction may play a key role in the disease progression. Unaffected relatives of patients with schizophrenia have higher rates of smoking, suggesting that physiological mechanisms driving smoking may also share a hereditary component similar to schizophrenia [18]. The majority of smokers (90%) start smoking before the onset of schizophrenia [17]. In adolescents who develop schizophrenia later in life, smoking rates are higher than in those who do not [19].

This evidence refutes the concept that rising smoking rates are due to a desire to relieve the consequences of schizophrenia symptoms. Higher smoking rates are linked to early disease onset, poorer quality of life, poorer prognosis, increased disease severity, and higher hospital admissions, signaling that the increased desire to smoke may be linked to important regulators of disease onset and progression [17,20-24]. Smoking has been linked to symptom relief in several early trials, suggesting that restoring a supposed nicotinic balance could improve illness prognosis significantly. Findings demonstrating a favorable impact of nicotine on different elements of cognitive performance have raised their interest in this regard [13,23]. A cohort study by Fang et al. evaluated the association between tobacco use and schizophrenia among 225 healthy controls and 244 schizophrenia patients [25]. Stratification analysis revealed that the rate of smoking was higher in male patients versus healthy controls, with male smokers having higher odds ratios for schizophrenia than non-smokers, despite the fact that there was no significant difference between schizophrenia patients and healthy controls in the entire sample.

Genetics of Smoking in Schizophrenia

Smoking is three times as prevalent in patients with schizophrenia compared to the general population [26]. While schizophrenia has several genetic risk factors, nicotine addiction is associated with numerous genes. The common genetic base (i.e., biological pleiotropy) of nicotine addiction and schizophrenia may explain why schizophrenia patients smoke so routinely and more severely compared to healthy controls [26,27]. Patients with schizophrenia who smoke do so to avoid either cognitive impairment and psychotic symptoms, or because smoking and schizophrenia share a genetic foundation, or perhaps smoking occurs before the onset of schizophrenia, making smoking a risk factor for schizophrenia [26]. A systematic analysis by Hu et al. detected 52 similar genes out of 331 genes were schizophrenia and 276 genes were nicotine addiction when they evaluated shared genes associated with both schizophrenia and smoking. The authors divided these shared genes for smoking and schizophrenia into multiple groups using network analysis, pathway analysis, and enrichment analysis. They discovered 12 significantly enriched pathways related to nicotine, cocaine, alcohol, and amphetamine addiction; serotonergic, dopaminergic, and glutamatergic synapse; neuroactive ligand-receptor interaction; cAMP signaling pathway; and estrogen signaling after performing pathway enrichment analysis [28]. Another study found pathways shared by schizophrenia and nicotine addiction associated with neural communication and neurotransmitter transduction, long-term potentiation, calcium signaling, and neuroactive ligand-receptor interaction pathway using large genome-wide association study datasets and Fagerström test for nicotine dependence in schizophrenia and concentration of plasma cotinine [26]. These findings suggested that nicotine addiction and schizophrenia may share a genetic relationship, that patients with schizophrenia smoke to relieve cognitive symptoms, or that nicotine addiction is a risk factor for schizophrenia [26]. Both studies suggested that assessing genes related to both polygenic disorders at a system level is necessary as nicotine addiction and schizophrenia could aid in the identification of shared genetic liability for both disorders, improve understanding of the relationship between the two disorders, and provide novel insights into the pathogenetic relationship between smoking and psychiatric disorders.

Nicotine and nAChR

Nicotine is a highly addictive chemical that aids in the start and continuation of tobacco usage [28]. The rewarding characteristics of nicotine are likely due to the rapid rate of transport to the brain after inhaling a cigarette puff. Nicotine acts primarily through nAChRs, which are ligand-gated ion channels composed of various pentameric combinations of three β subunits (β2-β4) and nine α subunits (α2-α10) organized around a central pore permeable to calcium, sodium, and potassium ions [29,30]. Most neuronal nAChRs in the central nervous system (CNS) are excitatory and fast-acting, control the release of other neurotransmitters such as norepinephrine, dopamine (DA), serotonin, glutamate, gamma-aminobutyric acid (GABA), and acetylcholine (ACh), and are found presynaptically [29,30]. However, the nAChRs on DA neurons in the ventral tegmental area (VTA), a key brain region for drug reinforcing, are situated postsynaptically. nAChRs are composed of homomeric receptors made up of a set of α subunits, or heteromeric receptors made up of a mixture of α and β subunits. The α4β2 and α7 subtypes of nAChRs are the most prominent in the brain [31].

The sensitivity of nAChRs to desensitization varies across subtypes. Glutamate-controlling nAChR subtypes, for example, desensitize more slowly than GABA-controlling nAChR subtypes [32]. Following extended nicotine exposure, this differential sensitivity to desensitization may result in higher glutamate release compared to GABA release. Increased DA release in the nucleus accumbens may result from a relative shortage of GABA over glutamate, which could be a key mechanism promoting tobacco use [32]. High nicotine doses or prolonged exposure result in antagonist-like nicotine activity as a result of nAChR desensitization [33]. Although the effects of nicotine on the upregulation and desensitization of nAChRs have been well documented, the roles these processes play in nicotine’s cognitive effects are complex and poorly understood. Nicotine appears to have an inverted J dose-response, with low doses or quick exposures benefiting cognitive function, and higher doses or protracted exposures either improving or impairing cognitive abilities [34]. The phasic and tonic activities of DA neurons are thought to mediate different aspects of goal-directed behavior; phasic activity facilitates cue-reward association and acquisition of incentive salience, whereas tonic activity is involved in response inhibition and behavioral flexibility [35]. The difficulty of clearly elucidating the mechanisms of nicotine’s effects on cognition is underscored by the varying brain expression patterns of nAChRs, their varying nicotine sensitivities, and the varying connections of nAChRs with other neurotransmitter systems that mediate cognitive function.

nAChR and Cognitive Function

Despite the fact that the neurobiological mechanisms behind the effects of nicotine on cognitive function remain unknown, new research at various levels continues to emerge. The hippocampal brain and prefrontal cortex regions have been linked to nicotine’s cognitive impacts. Moreover, it is possible that enhancing synaptic plasticity in particular brain circuits and improving signal-to-noise ratios improves cognition [35-37]. The nAChR subunits α2, α3, α4, α5, α7, β2, and β4 are likely to mediate the cognitive effects of nicotine, and, as mentioned below, the best evidence is for the implication of the α7 and β2 subunits [38,39].

α7 nAChR

α7 nAChRs generate ion channels that lack other nAChR subunits and have a high calcium permeability, allowing them to regulate the release of other neurotransmitters (e.g., glutamate) commonly found in the mammalian brain [33,40]. α7 nAChRs modulate synaptic plasticity similarly to NMDA-type glutamate receptors; however, they have a lower nicotine affinity than α4β2 nAChRs and do not desensitize at low nicotine doses [41,42]. These disparities in desensitization allow α7 nAChRs to remain active after α4β2 nAChRs have desensitized. Moreover, it has clinical therapeutic consequences, as explained below [43].

α7 nAChRs, which are abundant in the prefrontal cortex and hippocampus, have been demonstrated to influence numerous cognitive functions in both preclinical and human studies [44,45]. nAChR knock-out mice, for example, were shown to have more errors in sustained attention and working memory tasks than wild-type mice, according to multiple preclinical investigations [46,47]. However, the fact that these knock-out mice differ from wild-type mice in the density and distribution of other nAChR subtypes due to compensatory changes in the expression of other nAChR types during development makes interpretation of these data difficult [48].

A growing body of evidence suggests that α7 nAChRs are involved in cognitive deficiencies in various neuropsychiatric disorders, including Alzheimer’s disease, schizophrenia, Parkinson’s disease, and autism spectrum disorders [40]. α7 nAChRs, for example, have been linked to the sensory gating failure seen in schizophrenia [49-51]. Sensory gating helps to distinguish between irrelevant and significant stimuli, and it may be at the root of both the sensory overload and cognitive deficiencies seen in schizophrenic patients. In the postmortem brains of schizophrenic patients, the density of α7 nAChRs in the hippocampus, a region controlling sensory gating, is reduced, and reductions in α7 nAChRs have been linked to sensory gating impairment in schizophrenia [52-55]. Neural α7 nAChR expression and functioning deficits have been extensively associated with cognitive and early sensory gating impairments in schizophrenia patients and their relatives. Nicotine and α7 nAChRs agonists have also been demonstrated to correct sensory gating defects in animal models and schizophrenia patients [56]. Overall, these results pointed to a biological mechanism that could be tested to explain why people with schizophrenia smoke at such high rates [57-61]. Consequently, targeting α7 nAChRs with pharmacological agonists for the treatment of cognitive abnormalities in schizophrenia may have an indirect advantage of facilitating smoking cessation [40,62]. Noda et al. recently published a study that revealed α7 nAChR is linked to disease progression and is involved in the therapeutic impact of nicotine in schizophrenia [63].

Because α7 nAChRs are the common subtype of homomeric and heteromeric receptors containing α7 subunits are uncommon a short-hand for receptor classification is whether the receptor is an α7 nAChR or a non-α7 nAChR [64,65]. This can be determined pharmacologically by measuring sensitivity to α-bungarotoxin (α-BTX), a powerful and selective antagonist of the neuronal seven homomeric receptors in the brain. The α-BTX-sensitive receptors have a low affinity for nicotine and have quick kinetics, whereas α-BTX-resistant receptors have a higher affinity for nicotine, slower kinetics, are heteromeric, and are desensitized to low agonist doses [64,65].

β2 nAChRs

The β2 subunit is abundant in the basal ganglia, thalamus, and hippocampus, and growing evidence suggests that the β2 subunit plays a central role in the cognitive effects of nicotine [66]. Behavioral flexibility, working memory, inhibitory control, and attention are all compromised in β2 knock-out mice [67-70]. Furthermore, in β2 knock-out mice, nicotine delivery has no effect on associative memory performance compared to wild-type mice [71]. Nicotine therapy only partially compensates for two knock-outs’ deficiencies in exploratory behavior [72]. Finally, drugs that stimulate α4β2 nAChRs have been shown to improve cognition. For example, varenicline improves learning deficiencies caused by either alcohol or nicotine deprivation in mice or drug use, or nicotine withdrawal in mice, and improves cognition in humans [73,74].

Cognitive Effects of Nicotine

Several laboratory investigations have examined the effects of cigarette smoking or pure nicotine delivery on cognitive ability. The fact that cigarette smoke contains numerous other chemicals, in addition to nicotine, that may have cognitive-enhancing benefits complicates the interpretation of findings after smoking cigarettes [75,76]. Furthermore, the amount of nicotine delivered by smoking varies greatly depending on the type of cigarette used and individual smoking patterns. Many investigations on the modulation of cognition by nicotine have employed pure nicotine delivered through intravenous infusion, oral inhaler subcutaneous injection, transdermal patch, or nasal spray to overcome these constraints [77-82]. Smoking after a period of abstinence enhances cognition. Moreover, cigarette smoking, or nicotine, had robust cognitive-enhancing effects, according to a review of studies conducted until the 1980s [83]. However, it is unclear whether these cognitive-enhancing benefits were secondary to withdrawal relief rather than direct cognitive enhancement [83,84]. Heishman et al. conducted a meta-analysis of 41 placebo-controlled studies that included nicotine delivery to either non-smokers or satiated smokers to separate the direct cognitive benefits of nicotine from withdrawal alleviation [85]. Nicotine had a significant favorable influence on short-term episodic memory, fine motor, and working memory function, according to the researchers. Furthermore, both “alerting attention” (maintenance of an alert state) and “orienting attention” (directing attention to sensory events) were favorably influenced [86]. The effect of nicotine on cognitive performance was not dose-dependent, either within or across areas of cognitive function, showing the diverse nature of nicotine pharmacodynamics.

One widely held belief is that schizophrenia people smoke more cigarettes than the general population to “self-medicate” the cognitive deficiencies indicated above [80,81]. The effects of nicotine on cognitive performance in schizophrenia have been studied in numerous clinical trials [87]. The degree of nicotine dependency, nicotine satiety, nicotine withdrawal, and mode of delivery, on the other hand, varied significantly between studies. Gum, transdermal patch, orally and nasally inhaled nicotine, and subcutaneous nicotine have all been used in research to improve cognition in schizophrenia patients [88]. Boggs et al. conducted a systematic evaluation of studies in which nicotine was given to people with schizophrenia and concluded that nicotine may improve attention/vigilance acutely [88]. Unfortunately, due to the short duration of these studies, no long-term improvements to attention have been established. Furthermore, while the studies looked at various cognitive tests with a variety of outcomes, the vast majority of studies failed to account for multiple comparisons. The research implies that nicotine does not improve overall cognitive performance, especially in the long term.

Aside from these findings in satisfied smokers, research has demonstrated disparities in nicotine’s impact on cognitive processes in abstinent smokers and non-smokers. While nicotine increased working memory performance among abstinent smokers, it had no effect on non-smokers [89]. A double-blind, placebo-controlled study by Ettinger et al. reported that nicotine improved basic attentional capabilities in non-smokers in a study, but not response inhibition or top-down executive attentional function [90]. Another experimental study of overnight abstinent smokers found that smoking nicotine-yielding cigarettes increased overall accuracy in an attentional processing task when compared to placebo cigarettes, indicating that nicotine overcomes cognitive deficiencies caused by nicotine deprivation [91].

Furthermore, a recent study examined the effects of stopping and restarting smoking on cognition in schizophrenia patients to see if the self-medication theory was correct [92]. Cognitive abilities were assessed while smoking as usual (baseline), one week later (extended abstinence), one day after quitting (early abstinence), and three weeks after restarting smoking (resumption). To analyze different cognitive domains impacted by schizophrenia, researchers used tests of processing speed, executive function, verbal fluency, working memory, verbal memory, conflict resolution, and attention. With smoking cessation, resumption or abstinence, there were no significant differences in general cognitive performance. Consequently, the findings of this study cast doubt on the prevalent and long-held “self-medication” concept of smoking and schizophrenia, call into question the magnitude of smoking and nicotine’s pro-cognitive effects, and advocate for smoking cessation in people with schizophrenia [92]. Strategies for assisting smoking cessation include behavioral counseling to enhance motivation and support attempts to quit and pharmacological intervention to reduce nicotine reinforcement and withdrawal. Three drugs are currently used as first-line pharmacotherapy for smoking cessation, nicotine replacement therapy, bupropion, and varenicline.

The current review has some limitations. The studies included in this review examined the effects of a single dose of nicotine in the short term. As a result, projecting the effects of chronic tobacco use from the findings of these researches, which focused on acute nicotine effects found in an experimental context, is difficult.

## Conclusions

In schizophrenia, smoking is more common than in other populations. Nicotine has been shown to have cognitive-enhancing benefits in preclinical animals and human investigations, specifically in working memory, enhancement of fine motor control, attention, and episodic memory. The cognitive-enhancing properties of nicotine may play a role in cigarette smoking vulnerability, particularly in people with cognitive deficiencies, which include the majority of people with psychiatric disorders. Finally, future investigations are warranted to investigate the effectiveness of adjunctive nicotine agents on the preservation of cognitive and early sensory functions.
